# Effect of YuPingFeng granules on clinical symptoms of stable COPD: study protocol for a multicenter, double-blind, and randomized controlled trial

**DOI:** 10.1186/s12906-023-04271-7

**Published:** 2024-01-08

**Authors:** Ruifeng Chen, Yangqing Zhan, Zhengshi Lin, Xiao Wu, Jinchao Zhou, Zifeng Yang, Jinping Zheng

**Affiliations:** 1 The First Affiliated Hospital of Guangzhou Medical University, Guangzhou Institute of Respiratory Health, State Key Laboratory of Respiratory Disease, National Clinical Research Center for Respiratory Disease, National Center for Respiratory Medicine, Guangzhou, 510230 China; 2 Faculty of Chinese Medicine, Macau University of Science and Technology, Macau, 9998078 China; 3 Guangzhou Laboratory, Guangzhou, 510230 China

**Keywords:** YuPingFeng granules, COPD, SGRQ, Randomized controlled trial

## Abstract

**Background:**

Reducing current clinical symptoms and the risks of future exacerbations is the main goal of stable COPD management. Traditional Chinese medicine has unique advantages in chronic disease management. YuPingFeng (YPF), as a classical prescription, has been proven to reduce the risk of exacerbations, but there is a lack of high-quality evidence for the assessment of clinical symptoms and quality of life, particularly for the assessment of treatment response of microecology and immunity.

**Methods/design:**

This is a prospective, multicentre, randomized, double-blind, placebo-controlled clinical trial. A total of 316 eligible subjects with moderate to severe COPD will be randomized 1:1 to receive YPF or placebo. Participants will receive either YPF or a placebo at 5 g three times daily for 52 weeks. The primary outcome will be the change in the COPD Assessment Test (CAT) score after 52 weeks of treatment. Secondary outcomes will include changes in the St George's Respiratory Questionnaire (SGRQ) score and clinical symptom score, among others. Outcomes will be measured at each visit. The study will continue for 52 weeks and will include six visits to each subject (at day 0 and weeks 4,12,24,36 and 52). In the event of exacerbations, subjects will be required to go back to the hospital once on the first day of exacerbation or when their condition permits.

**Discussion:**

This trial will provide research methods to evaluate the clinical efficacy, safety, and the possible mechanism of YPF in the treatment of stable moderate-to-severe COPD patients. In addition, we hope to provide more possibilities for TCM to participate in the management of stable COPD.

**Trial registration:**

The trial was registered at the Chinese Clinical Trials Registry on 3 June 2022 (ChiCTR2200060476; date recorded: 3/6/2022, https://www.chictr.org.cn/).

## Background

Chronic Obstructive Pulmonary Disease (COPD) has become a major chronic disease that seriously endangers public health. The World Health Organization (WHO) predicted that the prevalence of COPD will continue to rise over the next 40 years and that by 2060 there will be more than 5.4 million deaths annually from COPD and related conditions [1, 2]. At present, the number of people with COPD has reached 99.9 million [3], making it the third leading cause of death in China and the second leading cause of disease burden in terms of disability-adjusted life years [4, 5]. Therefore, in order to reduce the incidence of acute exacerbations of COPD (AECOPD), improve the quality of life, reduce the mortality of patients, and reduce the social and economic burden, the treatment and management of stable COPD must be given attention. Currently, bronchodilators (β2 receptor agonists, anticholinergic drugs, combined bronchodilators, etc.), glucocorticoids, methylxanthine, etc. are commonly used drugs for the treatment of COPD [2]. However, while bronchodilators and glucocorticoids can relieve symptoms, they cannot effectively reduce oxidative stress or improve the downward trend in lung function [6–8]. In addition, COPD-related inflammation has a limited response to inhaled corticosteroids (ICS), and ICS alone do not prevent a sustained decline in FEV1 or mortality in COPD patients [9]. Long-term use of ICS is even associated with side effects, including osteoporosis, bleeding from peptic ulcers, hypertension, oral candidiasis, hoarseness, and pneumonia [9–12]. It can be seen that the commonly used drugs for the treatment of COPD have certain limitations and side effects, which create difficulties in the management of stable COPD. More importantly, no breakthrough new drugs have been approved for COPD, indicating that there is a huge unmet need in the pathogenesis of COPD and the development of preventive and trerapeutic drugs. In particular, safer and more effective preventive drugs need to be developed for earlier intervention, while making efficient use of scarce healthcare resources.

Traditional Chinese Medicine (TCM) is the greatest treasure trove waiting to be discovered by modern medicine. YuPingFeng (YPF), one of the classic TCM formulas with a tonic effect, is composed of Astragali Radix, Atractylodes Macrocephala Koidz, and Radix Saposhnikoviae. Recent pharmacological studies have shown that YPF can activate mucosal immunity, resists hormone-induced immunosuppression, and is recommended to be used in the treatment of stable COPD [13, 14]. A multicentre, randomized, double-blind, placebo-controlled clinical study we conducted confirmed that the combination of conventional treatment with YPF granules for 52 weeks significantly reduced the risk of acute exacerbations by 32.3% in moderate-to-severe COPD patients [15], but did not investigate the mechanism. Some small sample sizes or observational trials have shown that this drug may regulate immune function, improve lung function, and improves the quality of life [16, 17], but high-quality evidence-based evidence is still lacking. In recent years, the heterogeneity of AECOPD has been gradually recognized and preliminarily explored. The clinical phenotype differentiated by peripheral eosinophils is one of the important phenotypes of AECOPD. Eosinophils are generally considered to be a type of white blood cell associated with allergic diseases. In allergic disease, YPF has been shown to have good effects in both improving symptoms and controlling relapses [18–20]. A number of studies have also shown that YPF inhibits eosinophil activity and also inhibits eosinophil proliferation [21, 22]. We hypothesized that YPF may have a more pronounced clinical benefit on the eosinophilic phenotype of AECOPD. Therefore, we designed a prospective, multicentre, randomized, double-blind, placebo-controlled clinical trial based on eosinophil phenotypic stratification to evaluate the efficacy of YPF on clinical symptoms and quality of life, safety, and therapeutic response in microecology and immunity in stable COPD. We hoped to confirm the preventive and trerapeutic effects of YPF from different perspectives and to discover its possible mechanism.

## Method

### Study design

**Fig. 1:**
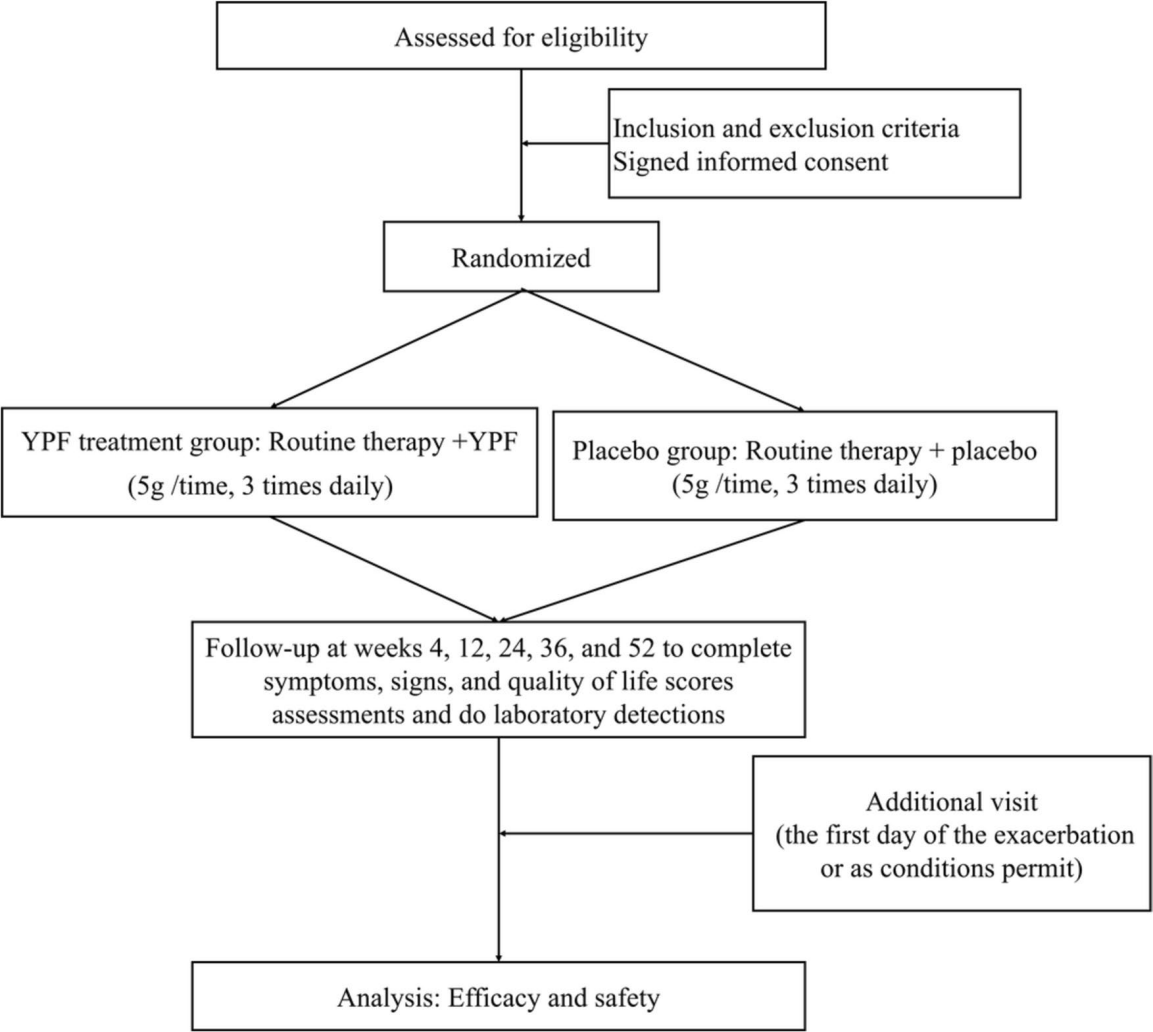
Flowchart of the trial design

This is a prospective, multicentre, double-blind, randomized controlled trial to evaluate the efficacy and safety of YPF granules in improving the clinical symptoms of stable COPD and to explore the underlying mechanism. Based on the exclusion and inclusion criteria, a total of 316 eligible subjects will be enrolled and randomly assigned to the YPF treatment group or control group in a 1:1 ratio. The study flowchart is shown in Fig. 1. Following informed consent and collection of baseline information, treatment will be administered for 52 weeks according to stratified block randomization. All subjects will return to the hospital once on day 0 and at weeks 4,12,24,36, and 52 to complete the assessment of their symptoms, signs, and quality of life scores (assessed by CAT, SGRQ, laboratory indicators, etc.). If subjects have an acute exacerbation, an additional visit will be made on the first day of the exacerbation or as conditions permit. The specific assessments at each visit are detailed in Table 1.

**Table 1:** Study schedule of specific assessments

	**Enrolment** **(Day -28 to 0)**		**Follow-up(± 3 days) **^**a**^					
Visit period	Screening stage ^c^	Visit 0	Visit 1	Visit 2	Visit 3	Visit 4	Visit 5	Additional visit ^**b**^
Visit time	Day -28 to 0	day 0	week 4	Week 12	Week 24	Week 36	Week 52	–
**Basic condition**								
Signing informed consent	●							
Demographic information ^**d**^	●							
General clinical information ^**e**^	●							
**Efficacy assessments**								
SGRQ scale		●	●	●	●	●	●	●
CAT scale		●	●	●	●	●	●	●
Symptom scale	●	●	●	●	●	●	●	●
Number of exacerbations and duration			●	●	●	●	●	●
Number of hospitalizations and reasons			●	●	●	●	●	●
Constitution of TCM^f^		●			●		●	
**Safety assessments**								
Vital sign	●	●	●	●	●	●	●	●
Physical examination	●		●	●	●	●	●	●
HCG	●							
Glycosylated Hemoglobin	●							
Lung function	●							
Blood routine	●				●		●	
NGHX	●				●		●	
Serum biochemicals	●				●		●	
Adverse events recorded		●	●	●	●	●	●	●
**Exploratory assessments **^**g**^								
Microecology of the respiratory tract		●	●	●	●	●	●	●
Herpesvirus infection		●	●	●	●	●	●	●
Classification and counting of lymphocytes		●	●	●	●	●	●	●
Inflammatory cytokines		●	●	●	●	●	●	●
Immunoglobulin		●	●	●	●	●	●	●
**Others**								
Starting time of treatment		●						
Combination therapy		●	●	●	●	●	●	●
Issue medications and diary cards		●	●	●	●	●		
Recycle medications and diary cards			●	●	●	●	●	
eCRF	●	●	●	●	●	●	●	●

*SGRQ* The St George's Respiratory Questionnaire, *CAT* COPD assessment test, *HCG* Human chorionic gonadotropin, *NGHX* Urine dry chemical analysis, *eCRF* Electronic case report form

^a^The time window of follow-up is ± 3 days, while the additional visit is ± 1 day;

^b^For additional visits, if subjects have an acute exacerbation, an additional visit will be taken on the first day of the exacerbation;

^c^The time window of screening is -28 ~ 0 days, and Day 0 is the day on which medication is started. During the screening period, the results of HCG, GHb, NGHX, lung function, blood routine, and serum biochemicals can be accepted within 7 days;

^d^Demographics, including age, gender, height, weight, marital status, ethnic origin, etc.;

^e^General clinical information, including medical history, course of the disease, number of exacerbations within the past year, etc.,

^f^Constitution of TCM [23]: based on the Classification and Determination of Constitution in TCM(ZYYXH/T157-2009) released by the China Association of Chinese Medicine;

^g^For the exploratory assessments, the chief unit is required to conduct tests every visit, while others are required on Day 0, Week 24, Week 52, and additional visits. Sputum, throat swabs, and blood samples are detected and analyzed by the central laboratory and the results are uniformly recorded, but not included in the CRF

### Sample size

According to the results of the previous study [15], the mean difference in CAT scores between the YPF group and the control group was 2.2, and the combined standard deviation was 6.2184. The sample size was calculated accordingly, with an α risk of 0.05 and a β risk of 0.2 in a two-sided contrast. This study required a minimum of 252 subjects. Allowing for 20% attrition, a total sample size of 316 subjects was required, 158 in the treatment group and 158 in the control group.

### Study sites and participant recruitment

Subjects will be recruited consecutively from eight university hospitals or hospitals with adequate experience of RCTs. These hospitals are located across China in the provinces of Guangdong, Yunnan, Shanghai, Guizhou, Shanxi, Hunan, and Xinjiang. The total recruitment period is planned to be 28 months, from 23/5/2022 to 24/9/2024. In order to screen suitable patients as quickly as possible, the eight participating hospitals will post recruitment advertisements on their websites and bulletin boards. The advertisements will include a brief description of the required subjects, study drugs, medical examinations, and how to participate in the trial. The First Affiliated Hospital of Guangzhou Medical University, as the lead institution, will recruit subjects first. Patients who meet the inclusion criteria but do not meet the exclusion criteria will be eventually be enrolled, and then treated and followed up according to the study protocol.

### Inclusion criteria

1. Aged 40 to 80 years, male or female;
2. Patients meet the diagnostic criteria for COPD (Guidelines for the diagnosis and management of chronic obstructive pulmonary disease (revised 2021 version) [24].)
3. Patients clinically diagnosed with moderate to severe COPD (based on the 2021 GOLD guidelines), with airflow limitation including the following: a postbronchodilator fixed ratio FEV1/FVC ≤ 70%, 30% ≤ FEV1 < 80% predicted;
4. The single score of expectoration ≥ 1 point;
5. Patients with a history of exacerbations in the past year: ≥ 2 moderate acute exacerbations or ≥ 1 severe acute exacerbation;
6. Patients were in stable condition in the past 4 weeks, without respiratory tract infection and AECOPD;
7. Volunteered to participate in the trial, and signed the written informed consent.

### Exclusion criteria

1. The interval between clinical diagnosis of COPD and enrolment in the trial is ≤ 12 months;
2. Patients needed long-term regular oxygen therapy, and oxygen inhalation for more than 15 h daily;
3. Patients have not previously received live attenuated vaccines, but plan to receive live attenuated vaccines during the trial;
4. Patients with lung transplantation or pneumonectomy, patients scheduled for lung transplantation or pneumonectomy, or patients enrolled in a pulmonary rehabilitation programme
5. Patients diagnosed with or with a history of asthma (according to the 2018 GINA guidelines or other recognized guidelines);
6. Patients with other significant lung diseases (such as pulmonary interstitial fibrosis, active tuberculosis, lung tumors, etc.);
7. Patients with severe heart diseases, liver, or kidney disease;
8. Patients with poorly controlled insulin-dependent diabetes mellitus (HBA1c ≥ 8.0%);
9. Immunocompromised patients;
10. Patients who have participated in clinical trials of other drugs within 3 months prior to enrolment or who are regularly taking proprietary Chinese patent medicine with the same efficacy as YPF.
11. Patients who are allergic to any components of the study drug;
12. Patients with serious neurological, hematological, gastrointestinal, cerebrovascular diseases, malignant tumors, and other current or previous serious diseases that could interfere with the study or require treatment that could interfere with the study;
13. Pregnant or breastfeeding women, and male patients planning to become fertile;
14. Patients considered by the investigator to be unsuitable for participation.

### Withdrawal and discontinuation criteria

All eligible patients have the right to withdraw from the trial at any time. During the trial, the following situations should be treated as withdrawals:

1. Serious complications, significant physiological changes, serious adverse events, and other serious events that occur during the trial.
2. Poor subject compliance of subjects (such as taking drugs in violation of the prescribed dose)
3. Subjects are unwilling to continue the trial and ask to withdraw from the trial
4. Refusal to accept medication or examination and lose of visitors.
5. Subjects become unblinded halfway through the trial.

The reasons for subjects’ withdrawal or discontinuation from the trial should be documented in detail. For those who withdrawn from the trial because of adverse events, the investigators should take appropriate treatment measures according to the subject’s actual situation, and try to complete the last test for subsequent statistical analysis.

### Interventions

All eligible patients will be randomized into two groups as follows:

1. YPF treatment group: YPF 5 g per time, three times daily, for 52 weeks.
2. Control group: placebo 5 g per time, three times daily, for 52 weeks.

YPF and placebo are uniformly supplied by Sinopharm Group Guangdong Medi-World Pharmaceutical Co., Ltd (Foshan, Guangdong Province, China). The experimental drugs, which have an identical appearance and almost similar taste, are manufactured according to the requirements of this clinical trial and meet the relevant quality requirements. YPF granules have been on the market for many years and there are clear standards for prescription, dosage and quality control. Details can be found in the 2020 edition of the Pharmacopoeia of the People’s Republic of China (hereafter referred to as the 2020 Chinese Pharmacopoeia). The placebo formulation consisted mainly of dextrin and mannitol (66.1% and 30.4% of the placebo, respectively). A small amount of 5% YPF extractum (2% of the placebo) was added to make the odour essentially identical to that of YPF. At the same time, a small amount of flavoring agents and food coloring was added to make the taste and appearance of the placebo similar to that of YPF.

Based on the clinical symptoms, signs and the GOLD guideline 2021, all patients in both groups will receive routine therapy (taking the commonly used maintenance medications in COPD, such as ICS/LABA/LAMA, theophylline, roflumilast, etc.) without intervention, but detailed medication should be recorded for summary analysis. At the same time, the medications (or other treatments) that have to be continually taken continuously for the underlying diseases, the dosage, frequency, and duration of use should also be recorded in the case report form (CRF). It’s forbidden to take other Traditional Chinese medicines or proprietary Chinese medicines with the same effect as YPF during the trial.

### Randomization, blinding and unblinding

The Interactive Web Response System (IWRS) will be used in this study. We will perform stratified block randomization for the eligible subjects, the absolute value of eosinophils in peripheral blood at the time of enrollment is taken as the stratification factor, and the cut-off value of the absolute value of eosinophils is set at 0.3 × 10^9^/L. The SAS 9.4 is used to generate the random number table and the drug random number table. Based on the two random number tables, the subjects are randomly divided into the YPF group and the control group. The administrator of IWRS will send the account number and password to the designated person in charge of each centre before the study. Then the investigator will log into the IWRS and enter the relevant information of the subject. The subject will be assigned a unique random number, which will be displayed by the IWRS.

In the trial, investigators, subjects, supervisors and clinical research coordinators will remain blinded. All experimental drugs and placebos are packaged in a uniform manner, with no difference in appearance, and blinded according to the drug random number table by people independent of the study. Investigators and subjects only know the random number and the drug number, and don’t know the treatment regimen represented by these random numbers.

Unblinding requirements: After all data have been entered into the Electronic Data Capture (EDC) system, the database is locked through a process of query, verification, and blinding review. The principal investigators and statisticians will then unblind the data and distinguish the groups of the subjects and corresponding interventions. The assessors then perform the statistical analysis after the data are unblinded. In case of an emergency (such as serious adverse events, serious complications, etc.) and the need for rescue measures, unblinding can be done urgently with the identification and signature of the principal investigator. The sponsor and the principal investigator should be notified within 24 h of the emergency unblinding, and the reason for the emergency unblinding should be explained. The rate of emergency unblinding should not exceed 20%, otherwise, the study may be considered a failure. Unblinding should not be performed in cases of withdrawal for therapeutic reasons.

### Outcome measurements

#### Efficacy indicators

The primary outcome is the changes in the COPD assessment test (CAT) score after 52-week of treatment. Specifically, the differences between the total CAT score at week 52 and baseline will be calculated respectively for the experimental and the control groups, respectively, and the difference between the two groups will be compared. The secondary outcomes include the following eleven indicators:

1. Annualized rates of moderate or severe COPD exacerbations. The measure is the number of acute exacerbations of moderate or severe COPD during 52-week treatment/total participants in the group. The definition of an acute exacerbation of COPD (AECOPD): is an acute change in a patient’s baseline dyspnea, cough, and/or sputum beyond day-to-day variability sufficient to warrant a change in therapy. The severity of AECOPD is graded as follows: (1) Mild: requires only increased treatment with short-acting bronchodilators, such as short-acting β-receptor agonists (SABA); (2) Moderate: requires increased antibiotics and/or oral glucocorticoid hormone; (3) Severe: requires hospitalization or emergency treatment, may be complicated by acute respiratory failure.
2. All-cause admission rate and admission rate due to acute exacerbation. All-cause admission rate = (total number of admission during 52-week treatment)/ (total number of participants in the group) and the admission rate due to acute exacerbation = (number of admission due to acute exacerbation during 52-week treatment)/ (total number of participants in the group).
3. The changes in the St George's Respiratory Questionnaire (SGRQ) score.
4. The proportion of patients with an SGRQ response (defined as an improvement of ≥ 4 units from baseline).
5. The changes in the CAT score.
6. The median time to the first exacerbation of COPD and the median time to the first exacerbation of moderate or severe COPD during treatment.
7. Duration and time interval of acute exacerbations compared with the control group.
8. Annualized rates of acute exacerbations of COPD of different severity.
9. The changes in clinical symptom score (Table 2) compared with the control group.
10. The changes in the individual clinical symptom score compared with the control group.
11. Frequency of first-aid drug use.
12. The changes in TCM constitution from the baseline to week 24 and week 52.

**Table 2:** The clinical symptom assessment scale

Item	Symptoms Assessment rating scale			
	0 (none)	1 (mild)	2 (moderate)	3 (severe)
Cough	No	Intermittent cough during the day does not affect work and life	Cough during the day or occasionally cough at night, slightly affecting work	Frequent cough, affecting work and rest
Cough sound	No	Low coughing sound	Weak coughing sound	Weak and low coughing sound
Expectoration	No	Sputum volume:10-50 ml	Sputum volume:51-100 ml	Day and night expectoration, Sputum volume > 100 ml
Dyspnea (difficulty breathing)	No	Difficulty breathing when walking or walking upstairs	Difficulty breathing with slight movement	Difficulty breathing occurs at rest
Pant	No	Occasional, does not affect work and life	Visible day and night, slightly affecting work	Can’t lay down, affecting sleep and activity
Mental weariness	No	in low spirits	mental fatigue	spiritual malaise
Fatigue	No	Feeling tired when labor	Feeling tired when moving	Getting tired even if not moving
Lack of qi and no desire to speak	No	Lack of qi, and don’t like talking too much	Lack of qi, and not enthusiastic about talking	Have no strength, don't even want to speak
Sleep disturbance	NO	easy to wake up and wake up early in the morning, but does not affect normal work	sleep less than 6 h per day, difficult to maintain normal work	sleep less than 4 h a day, difficult to maintain normal work

#### Safety evaluation

The safety assessment will include the following: (1) Vital signs (body temperature, heart rate, breathing, and blood pressure) will be measured at each visit. (2) Laboratory tests including routine blood and urine tests, liver and kidney function tests, electrocardiogram (ECG), and other inspections will be performed once at baseline, 24 weeks, and 52 weeks. (3) Adverse events will be evaluated and recorded on the case report form (CRF).

#### Exploratory indicators

The exploratory assessment will include the following: (1) Inflammatory indicators (including but not limited to IFN-γ, IL-6, IL-1β, TNFα, etc.) and lymphocyte classification and count. (2) Microecology of the respiratory tract, mainly to detect the microecological diversity (α diversity, β diversity) and its dominant flora. (3) The infection condition of herpes virus (Herpesvirus, Cytomegalovirus, Epstein-Barr virus). (4) Immunoglobulin (IgA, IgM, IgG).

### Adverse events (AEs)

AEs are any adverse medical events that occur after treatment and are manifested as unexpected symptoms, signs, diseases, or abnormalities in laboratory results. During the trial, AEs are mainly assessed by safety examination and investigator neutral interrogation. AEs are then recorded in detail on CRFs, including the severity, time of onset (time of symptom onset), duration and time of termination, the use of combinations and experimental drugs, effective measures and outcomes, etc. According to the Consensus of Expert on Safety Evaluation of Drug Clinical Trial in Guangdong (Version 2020), determine the causal relationship between the AE and the experimental drug, and divide the correlation evaluation into six levels: definite, probable, possible, possibly irrelevant, to be evaluated and impossible to evaluate.

### Quality control and data management

To ensure the successful process of the study and the reliability of the conclusions, the clinical physicians and nurses directly involved in the study all keep a GCP training certificate and are trained in some specific course before the trial. Guangzhou Evidence-Based Medicine Tech Co., Ltd (Guangzhou, Guangdong province, China) as the contract research organization (CRO) is commissioned to make a detailed clinical monitoring plan (CMP) according to the trial. The CMP includes the specific clinical research associate (CRA), frequency, degree, report of the monitoring, etc. The CRA will complete the tasks according to the CMP and the actual progress.

Clinical Trial Electronic Data Capture System (EDC) will be used in this trial and all data will be entered into the EDC system by trained and qualified investigators. If there is any doubt about the data in the EDC system, the data manager will issue an inquiry to the investigator to make sure the data is true and correct. Once the data have been reviewed and verified to be correct, the database is locked by the principal investigator, sponsor statistical analyst, and the data manager. In general, a locked database cannot be unlocked.

### Statistical analysis

The SAS 9.4 statistical software will be used for statistical analysis. Both full analysis set (FAS) and per-protocol set (PPS) will be used for baseline and efficacy analysis, while the safety set (SS) for the safety analysis. Data will be descriptively analyzed and presented as means and standard deviations, medians and interquartile ranges, and frequencies. The minimum, maximum, P25, median, and P75 are given when necessary. All analyses will be two-sided, with a *p*-value of less than 0.05 considered statistically significant.

#### Demographic and baseline analysis

T-test or non-parametric statistical methods will be used for quantitative data according to the distribution of variables. The χ2 test or Fisher's exact probability test will be used for qualitative data, and the Wilcoxon rank-sum test will be used for ranked data.

#### Efficacy analysis

Different analysis methods will be adopted according to the variable type of efficacy indicators. Specifically, Fisher's exact test will be used to compare categorical variables, including the annualized rates of moderate or severe COPD exacerbations, all-cause admission rate, admission rate due to acute exacerbation, the proportion of patients with an SGRQ response, annualized rates of acute exacerbation of COPD with different severity, and the frequency of first-aid drug use. The duration and interval of AECOPD will be tested by the log-rank test, and the Cox proportional-hazards model will be used to calculate the hazard ratio (HR) and 95% confidence interval (CI). The changes in the CAT score, the SGRQ score, the clinical symptom score, and the single clinical symptom score will also use the log-rank test and the Cox proportional-hazards model for analysis.

#### Safety analyses

All AEs occurring during this study will be listed and the incidence of AEs will be calculated. The χ2 test or Fisher's exact test will be used to compare the incidence of AEs among groups. As for the laboratory indexes, the frequency table and specific situation of changes will be listed.

#### Subgroup analyses

The absolute value of blood eosinophils will be used as a stratification factor to perform the stratification analysis of the efficacy indicators. The efficacy indicators will also be analyzed by TCM constitution, if possible. The assessment of TCM constitution is based on the Classification and Determination of Constitution in TCM(ZYYXH/T157-2009) released by the China Association of Chinese Medicine.

### Safety and ethics

YPF has been used clinically for many years and has good safety in the preliminary study [15]. To ensure the safety of subjects, all subjects are given YPF or placebo treatment in addition to routine therapy and will be received relevant safety checks. Investigators will also take timely symptomatic treatment of AEs in the subjects. Before enrollment, investigators will inform the subjects about the details of the trial, including the purpose, methods, procedures, possible benefits, and risks, and answer any questions the subjects may have. Subjects will not perform any study procedures until they have voluntarily signed the written informed consent form. They can withdraw from the trial at any time without damage during the trial. If the protocol or informed consent is significantly modified, we will re-submit the ethics for approval and reacquire the consent of the subjects again.

### Dissemination

No data will be provided to any third party in any form without the written consent of the sponsor. All data and analysis will remain blinded until the results are published. The final results will be published in relevant journals in the form of literature.

## Discussion

The management of stable COPD is a complex clinical challenge, and the 2022 GOLD guideline states that the aim of management is to reduce both current clinical symptoms and the risks of future exacerbations. In particular, AECOPD directly poses a serious threat to life. In our previous clinical study [15], we confirmed that YPF reduces the risk of AECOPD, but there was a lack of high-quality evidence of symptom reduction and quality of life improvement. COPD is a progressive and debilitating respiratory disease, characterized by dyspnea and irreversible airflow restriction. Most COPD patients experience various symptoms at the same time, such as dyspnea, sleep disturbance, fatigue, etc. At the same time, different symptoms synergistically damage the quality of life, functional status, and psychological status of patients. Among them, 64.2% of COPD patients report that dyspnea impaired their quality of life, and COPD patients with depression, anxiety, and fatigue show the poorer quality of life [25]. In this trial, we design a clinical symptom assessment scale including cough, expectoration, fatigue, etc., to standardize the evaluation of each common clinical symptom of COPD. At present, the SGRQ is considered to be the most effective scoring system for evaluating quality of life in COPD patients. However, the conversion method of the SGRQ is complex, and the content is numerous and time-consuming, making it difficult for some patients to complete independently. The CAT is simple in structure, easy to operate, and short in time, which is more suitable for patients with limited education and low compliance than SGRQ. So we are using the two questionnaires in this trial, with the changes in the CAT score after 52-week of treatment as the primary outcome, and the clinical symptoms and the SGRQscores as the secondary outcomes, and we hoped that the results will be mutually confirming and complimentary.

Microbiota is defined as the “ecological community of commensal, symbiotic and pathogenic organisms that share our body space” [26]. In recent years, with the application of various new techniques (such as NGS) to detect microbes, increasing evidence indicates that respiratory and intestinal microbiota and their associated mucosal immunity are related to the clinical manifestations, acute exacerbations, and prognosis of COPD [27–32]. Studies have showed that the pulmonary microbiota also changes during AECOPD compared to stable condition samples [33–37]. Pulmonary microbial composition was transferred to *Proteobacteria*, while Firmicutes decreased [33]. Manifested by an increase in *Hemophilus influenza* and a decrease in the relative abundance of *Streptococcus pneumonia* species [34]. In addition, there was a significant increase in Moraxella catarrhalis, and this species was positively correlated with the percentage of neutrophils in sputum, suggesting the possibility of interaction between host immune response and microbiome [33]. A case–control study showed that there was a strong and independent relationship between the presence of peptic ulcer disease and lung function indices in COPD patients with peptic ulcer disease [30]. Animal studies have indicated that gut microbiota and high-fiber diets play a role in the pathogenesis of COPD in smoking-exposed mice [38, 39]. Therefore, in this study, biological samples including blood and sputum will be collected to evaluate the therapeutic response of YPF in stable COPD from the perspective of microecology and immunity, and to explore the mechanism of YPF in reducing the risk of AECOPD.

In this study, we will also perform stratified analysis. Firstly, clinical studies have indicated that peripheral blood eosinophils are a biomarker of exacerbation risk in patients with a history of exacerbations and may be useful in guiding inhaled corticosteroids (ICS) treatment in patients at high risk of AECOPD at stable stage [40, 41]. It was also confirmed that YPF had resistance to hormone-induced immunosuppression. Therefore, stratification based on absolute values of blood eosinophils can explore whether there is a difference in the clinical efficacy of YPF on COPD eosinophilic phenotype and non-eosinophilic phenotype, which is instructive for YPF combined with hormonal treatment of COPD. Secondly, as YPF is a classical prescription, this study will conduct a stratified analysis by TCM constitution, which is in line with the clinical practice of TCM use. This study is a randomized, double-blind, placebo-controlled, multicenter clinical trial with a long intervention duration of 52 weeks, which is also consistent with the characteristics of TCM intervention for chronic diseases. However, long-term medication might affect patient compliance, and the collection of biological samples has time requirements, which requires high compliance. Second, the study included patients with moderate-to-severe COPD, which may not represent all COPD patients, especially those with early COPD. Whether YPF is suitable for the health management of COPD requires further clinical evaluations in different COPD populations.

## Data Availability

Not applicable.

## Abbreviations

COPD: Chronic obstructive pulmonary disease
WHO: World Health Organization
AECOPD: Acute exacerbation of COPD
TCM: Traditional Chinese medicine
YPF: YuPingFeng
SGRQ: The St George's Respiratory Questionnaire
CAT: COPD assessment test
HCG: Human chorionic gonadotropin
NGHX: Urine dry chemical analysis
eCRF: Electronic case report form
SAS: Statistical Analysis System
ECG: Electrocardiogram
FAS: Full analysis set
PPS: Per-protocol set
SS: Safety set
AE: Adverse event
CRO: Contract research organization
CMP: Clinical monitoring plan
CRA: Clinical research associate
EDC: Clinical trial electronic data capture system
