# Correction: Effect of YuPingFeng granules on clinical symptoms of stable COPD: study protocol for a multicenter, double-blind, and randomized controlled trial

**DOI:** 10.1186/s12906-024-04369-6

**Published:** 2024-02-05

**Authors:** Ruifeng Chen, Yangqing Zhan, Zhengshi Lin, Xiao Wu, Jinchao Zhou, Zifeng Yang, Jinping Zheng

**Affiliations:** 1 The First Affiliated Hospital of Guangzhou Medical University, Guangzhou Institute of Respiratory Health, State Key Laboratory of Respiratory Disease, National Clinical Research Center for Respiratory Disease, National Center for Respiratory Medicine, Guangzhou, 510230 China; 2 Faculty of Chinese Medicine, Macau University of Science and Technology, Macau, 9998078 China; 3 Guangzhou Laboratory, Guangzhou, 510230 China

**Correction: Complement Med Ther 24, 25 (2024)**

10.1186/s12906-023-04271-7

Upon publication of the original article [1], it was noticed that the author “Ruifeng Chen” was mistaken for the co-corresponding author of the article, and the author “Yangqing Zhan” was mistaken for the co-author. The authors “Ruifeng Chen” and “Yangqing Zhan” were both the first authors. The authors “Zifeng Yang” and “Jinping Zheng” were the co-corresponding authors.

The original article has been corrected.
